# Characterization of the Bell-Shaped Vibratory Angular Rate Gyro

**DOI:** 10.3390/s130810123

**Published:** 2013-08-07

**Authors:** Ning Liu, Zhong Su, Qing Li, MengYin Fu, Hong Liu, JunFang Fan

**Affiliations:** 1 School of Automation, Beijing Institute of Technology, Beijing 100084, China; E-Mails: liuning1898@bit.edu.cn (N.L.); fumy@bit.edu.cn (M.F.); kalmanliuhong@126.com (H.L.); 2 Beijing Key Laboratory of High Dynamic Navigation Technology, Beijing Information Science & Technological University, Beijing 100101, China; E-Mails: liqing@bistu.edu.cn (Q.L.); wyhffjf@gmail.com (J.F.)

**Keywords:** bell-shaped vibratory angular rate gyro, BVG, coriolis vibratory gyro, bell-shaped resonator

## Abstract

The bell-shaped vibratory angular rate gyro (abbreviated as BVG) is a novel shell vibratory gyroscope, which is inspired by the Chinese traditional bell. It sensitizes angular velocity through the standing wave precession effect. The bell-shaped resonator is a core component of the BVG and looks like the millimeter-grade Chinese traditional bell, such as QianLong Bell and Yongle Bell. It is made of Ni43CrTi, which is a constant modulus alloy. The exciting element, control element and detection element are uniformly distributed and attached to the resonator, respectively. This work presents the design, analysis and experimentation on the BVG. It is most important to analyze the vibratory character of the bell-shaped resonator. The strain equation, internal force and the resonator's equilibrium differential equation are derived in the orthogonal curvilinear coordinate system. When the input angular velocity is existent on the sensitive axis, an analysis of the vibratory character is performed using the theory of thin shells. On this basis, the mode shape function and the simplified second order normal vibration mode dynamical equation are obtained. The coriolis coupling relationship about the primary mode and secondary mode is established. The methods of the signal processing and control loop are presented. Analyzing the impact resistance property of the bell-shaped resonator, which is compared with other shell resonators using the Finite Element Method, demonstrates that BVG has the advantage of a better impact resistance property. A reasonable means of installation and a prototypal gyro are designed. The gyroscopic effect of the BVG is characterized through experiments. Experimental results show that the BVG has not only the advantages of low cost, low power, long work life, high sensitivity, and so on, but, also, of a simple structure and a better impact resistance property for low and medium angular velocity measurements.

## 1. Introduction

The bell-shaped vibratory angular rate gyro (abbreviated as BVG) is a novel shell vibratory gyroscope, which is inspired by the Chinese traditional bell. It senses angular velocity through the standing wave precession effect. The Bell-shaped resonator is a core component of the BVG and looks like the millimeter-level Chinese traditional bell, such as QianLong Bell and Yongle Bell. BVG has not only the advantages of low cost, low power, long work life, high sensitivity, and so on, but also a simple structure and good anti-impact performance for low and medium velocity angular measurements [1–4].

The classic vibratory shell solid wave gyro includes: a hemispherical resonator gyro, a cylinder gyro, a ring vibratory gyro, a disc plant vibratory gyro, and so on [5,6]. Those are high accuracy mechanical gyros, which have a high quality factor (Q factor). At present, the HRGis the highest accuracy of coriolis vibratory gyros and has many mature products. It is widely used for aircraft navigation, strategic accuracy systems, oil borehole exploration, planetary exploration, *etc.* [7,8]. HRG has attracted many researchers, such as Lynch and Shatalov, about modeling the theory, processing the signal, analyzing the error, *etc.* [9–11]. The future development direction of HRG will be achieved through micromechanics [12]. The cylinder vibratory gyro is designed by Innalabs, Waston, BAE, and is widely used for some low and medium precision occasions, such as oil borehole exploration and vehicle navigation [13,14]. There are many researchers researching this. For example, Loveday analyzed the error of the cylinder vibratory gyro and presented an analysis method about the imperfect cylinder resonator [15]; Kristiansen researched the nonlinear model of the cylinder resonator and presented a control method and observations [16]; Wu Xuezhong analyzed the character of resonator vibration, temperature and error and presented a method of error compensation [17,18]. A ring vibratory gyro is widely used in MEMSand has many types. However, the accuracy is lower and lower than HRG [19–22]. The hemispherical resonator of HRG is made of quartz, which does not bear high impact. Besides, HRG used electrostatic excitation and capacitance detection, which are complex and expensive. The cylinder vibratory gyro is made of metal and uses the piezoelectric element to excite and detect. However, the impact resistance property, accuracy and vibration stability are restricted by the structure of the cylinder. Pointing to this condition, Innalabs uses the quartz to fabricate the resonator. In this way, the cylinder vibratory gyro improves accuracy and stability. However, the impact resistance property and cost are influenced by the material [23]. In conclusion, a high impact resistance, low cost, high performance gyro is in great need for the current market.

The proposed bell-shaped vibratory angular rate gyro is inspired by the traditional Chinese bell. Changing the resonator structure of the traditional vibratory gyro improves accuracy and impact resistance. There are many characteristics of the traditional Chinese bell, which includes beautiful sound, vibration stability, a steady mode shape, good impact resistance, *etc.* Therefore, the project group decided to use the bell structure to make the vibratory gyro, which is named the bell-shaped vibratory angular rate gyro; the resonator is bell-shaped. It is made by a constant modulus alloy, using piezoelectricity to excite and detect. This improves the impact resistance property and maintains the advantages of low cost and high performance. It is desired that the bell structure improves the performance of the gyro. For the novel structure of the gyro, the project group published an article, which describes the design procedure and preliminary experimentation and does not deeply analyze the theory of the BVG. Besides, the angle of the standing wave precession is detected through a capacitance sensor [24]. In the 1970s, Lynch and other researchers proposed a bell gyro. It is the same as a hemispherical resonator gyro, different from the BVG [25–27]. Since then, there has been no other resource published.

There are many problems using a bell structure to make a gyro. It is difficult to analyze the bell-shaped resonator, because the vibratory character is very complex and the middle surface is not described using the single linear function. To summarize, the revolution vibratory shell finds a method to solve the vibratory problem of a bell-shaped resonator. For the HRG, VA Matveev and other researchers used the theory of a thin shell to analyze the character of a hemispherical resonator. He presents the equilibrium differential equation and derives the governing equation [1]. This method is an important method for HRG, currently. Shatalov, Kristiansen and others used the Hamilton principle to analyze the character of the HRG and presented a simplified equation [10,16]. Leissa and others used the Ritz-Rayleigh analysis method to research the character of many types of revolution shell in the vibration control field. However, it only analyzes the natural frequency based on assuming the mode shape [28,29]. For the bell-shaped resonator, it chooses the thin shell theory to solve the problem. Others methods have many hypotheses that are not appropriate for a bell-shaped resonator [1,30]. The structure of a bell-shaped resonator chooses the parabolical structure. It is convenient to analyze and model. Based on the structure, this paper analyses the vibratory character of a bell-shaped resonator and designs the prototypal gyro.

In this article, we explore bell-shaped resonator structure and present the design, analysis and experimentation of the BVG. The important work is as follows: to design the structure of a bell-shaped resonator and present the principle of the work, shown in Section 2. In Section 3, a theoretical analysis of the proposed resonator is performed using the theory of thin shells. The strain equation, internal force and resonator's equilibrium differential equation are derived in the orthogonal curvilinear coordinate system. On this basis, the mode shape function and the simplified second order normal vibration mode dynamical equation are obtained. In Section 4, the coriolis coupling relationship about the primary mode and secondary mode is established. The method of the signal processing of the BVG and the control loop of the prototype are presented. In Section 5, to analyze the impact resistance property of the bell-shaped resonator, which is compared with other shell resonators using the Finite Element Method, demonstrates that the BVG has the advantage of better impact resistance. In Section 6, a reasonable method of installation and a prototypal gyro are designed. The gyroscopic effect of the BVG is characterized through experimentation. Experiment results show that the BVG has not only the advantages of low cost, low power, long work life, high sensitivity, and so on, but also a simple structure and better impact resistance for low and medium angular velocity measurements. Finally, some important conclusions are drawn.

## 2. Design Concept

### 2.1. The Structure of the Gyroscope

**Figure 1.**
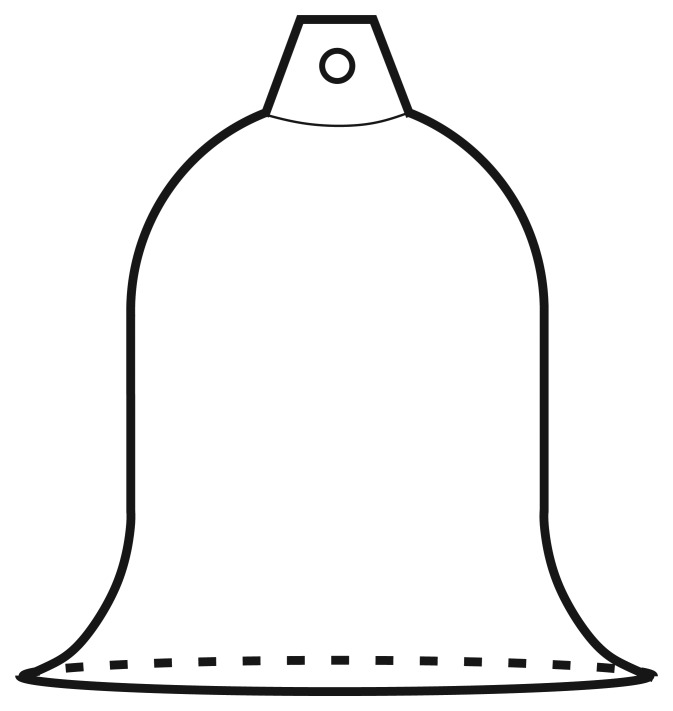
Schematic of a traditional Chinese bell.

**Figure 2.**
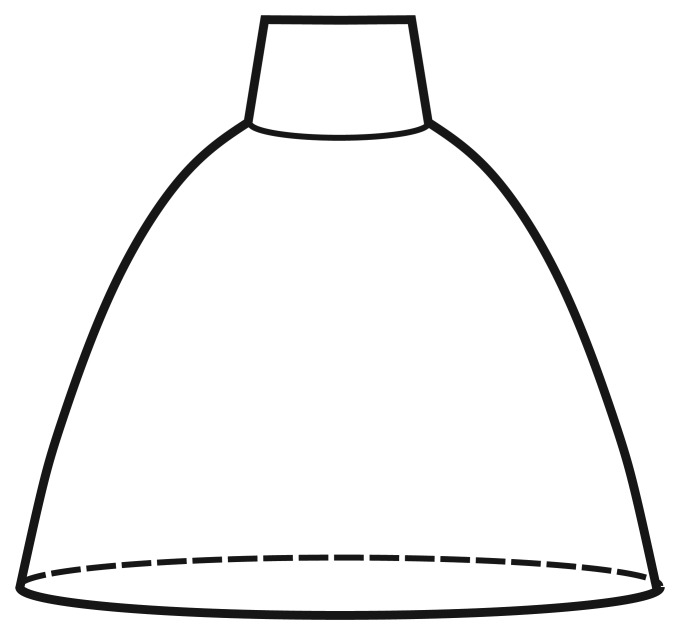
Schematic of a parabolic structure.

The core component of the BVG is a bell-shaped resonator, which influences the performance of the gyro directly. The key of the BVG is the structure of the bell-shaped resonator. The bell-shaped resonator is inspired by the traditional Chinese bell, as shown in Figure 1. As far as we known, there are many characteristics of traditional Chinese bells that includes beautiful sound, vibration stability, steady mode shape, high impact resistance *etc.* The technology of multiple surface fusions is very important. However, it has not been explained clearly until now [31]. It is convenient to describe a bell-shaped resonator using the revolution parabolic structure, which maintains the vibratory character of the traditional bell, as shown in Figure 2.

**Figure 3.**
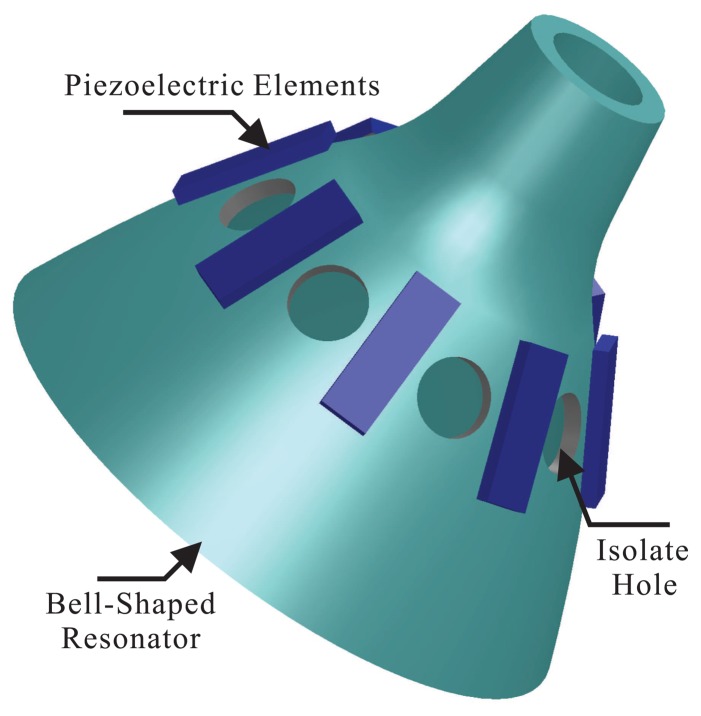
Schematic of the resonator.

The bell-shaped resonator looks like the traditional bell, which is designed by a parabolic structure intuitively. In order to reduce the cost and design procedure, the structure of the top, middle and bottom merge into a parabola, and this is achieved to integrate the design. This merge does not change the vibratory character of the structure. The position of the excitation electrode and the detection electrode is very important for the BVG. For the actual bell, to excite bell vibration, one uses a stick to hit the wall of the bell. The vibration effect is very significant. However, the bell-shaped resonator selects the position on the wall of bell. In order to ensure the power consumption and cost of the gyro, it controls the mode shape and detects the precession of the standing wave based on piezoelectricity. The piezoelectric elements are attached to the wall of the bell-shaped resonator to excite the primary mode and detect the second mode. Using the isolated hole reduces the disturbance between neighboring electrodes, as shown in Figure 3.

### 2.2. Working Principle

**Figure 4.**
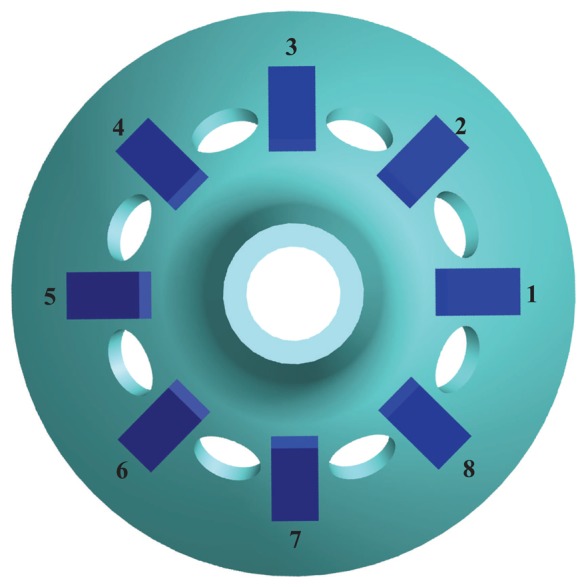
Schematic of mount electrode.

**Figure 5.**
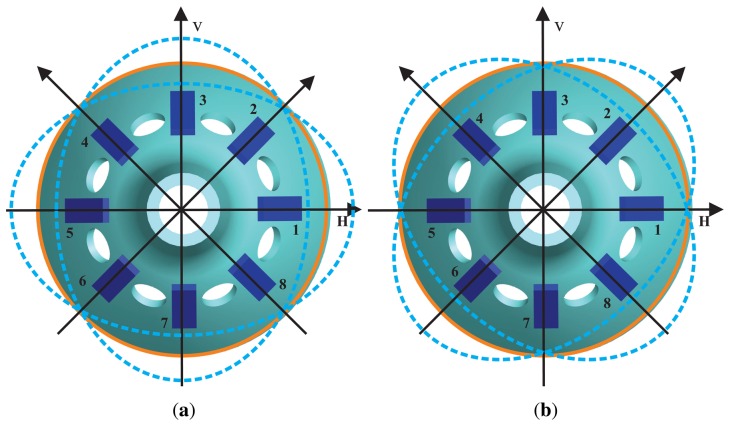
Schematic of the working principle. (**a**) Primary Mode. (**b**) Second Mode.

**Figure 6.**
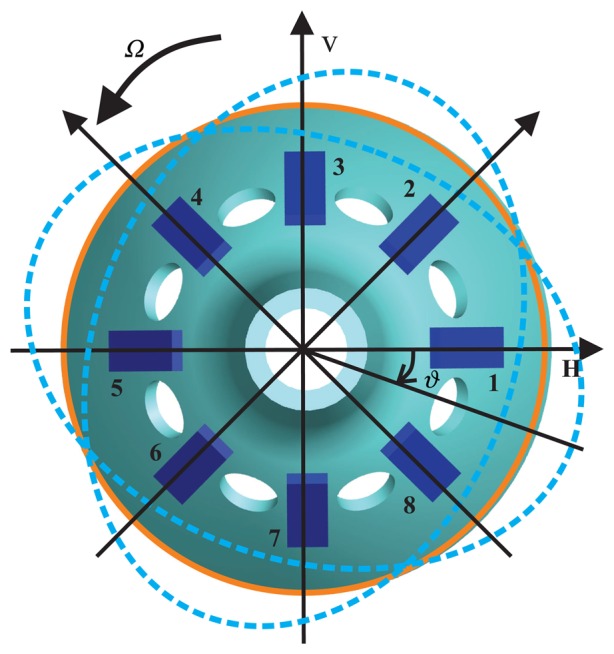
Schematic of the standing wave precession.

The BVG sensitizes angular velocity through the standing wave precession effect. The eight piezoelectric elements are attached to the wall of the bell-shaped resonator uniformly, as shown in Figure 4. Based on the inverse piezoelectric effect, excitation electrodes excite the bell-shaped resonator, and the resonator produces a work mode, which is named the primary mode or the excite mode, shown in Figure 5a. For the vibration shell, the same work frequency has two types of modes, which are different, 45°. The other mode is named the second mode or sensitive mode, shown in Figure 5b. The coriolis couples them. The amplitude of the second mode is proportional to the input angular velocity and produces the standing wave precession. In Figure 6, when the angular velocity is applied to the axis of symmetry counterclockwise, the sanding wave angle changes. The precession angle is –*ϑ* and proportional to the frame rotation angular velocity.

During practical application, the stress wave propagates on the resonator, causing the standing wave, and the piezoelectric elements sensitize the stress wave and solve the angular velocity. In order to reduce the influence of piezoelectric elements when the resonator is rotated and to improve the excitation efficiency, the elements should be set near the constrained boundary. Such an excitation type is like the principle of leverage, using a small amount of force to achieve a large deformation to improve the excitation efficiency.

**Figure 7.**
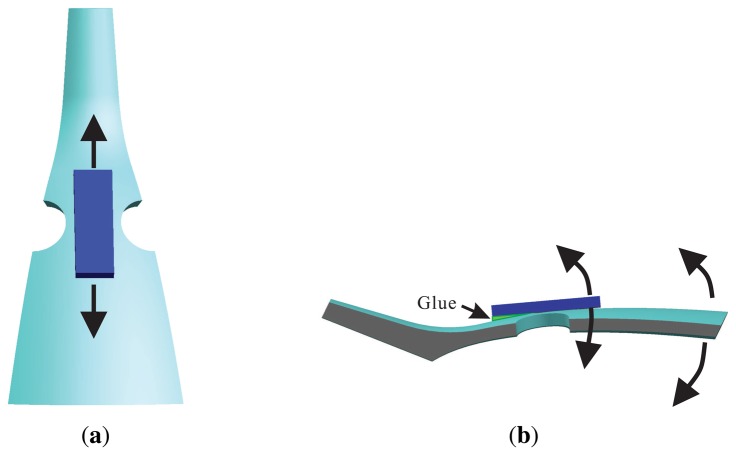
Schematic of piezoelectric work principle. (**a**) Contract and Expand Force. (**b**) Bend Force.

The piezoelectric elements chose the PZT5A, which was polarized in the thickness direction. The first and fifth elements contract and expand while applying an alternating current signal (in Figure 7a). When the top of the elements are bounded, the contraction and expansion force will transfer to the bend force (in Figure 7b) and excite the shell vibration. The piezoelectric elements and resonator are attached together by conductive adhesives. Controlling the painting procedure makes a rigid connection between them. The one pole of elements connected to the resonator is the GND, the others are the signal of the input or output. The piezoelectric element senses the vibratory signal based on the piezoelectric effect. Using the third and seventh element feedback signals and the first and fifth excitation elements, standing wave steady control is achieved. The control loop includes an amplitude control loop and a frequency control loop. With particular emphasis, the work mode of the BVG is the force-rebalance mode [15,18]. The second and sixth elements sensitized angle of precession is used as the controller input to drive the fourth and eight elements to restrain the precession of standing wave. The amplitude of restrain variable is proportional to the angular velocity and is used as the output signal of the BVG after calibration.

## 3. Modeling and Simulation

For the shell vibratory gyro, the analysis of the resonator vibratory character is very important and focuses on the mode shape function, precession factor and natural frequency. The BVG is a novel structure in the vibratory gyro field and has a unique vibratory character. The analysis of internal force distribution and the equilibrium differential equation are presented using the theory of thin shells. It derives the simplified second normal vibration mode dynamic equation based on the parameters of the gyro.

### 3.1. Coordinate System

**Figure 8.**
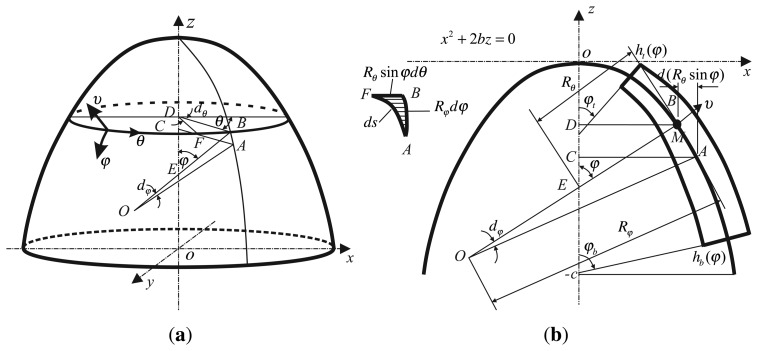
Schematic of the working principle. (**a**) Orthogonal Curvilinear Coordinate System. (**b**) Cross-Section Schematic.

The bell-shaped resonator is a parabolical structure and described in the orthogonal curvilinear coordinate system (*φ*, *θ*, *v*), shown in Figure 8[32,33]. In the Cartesian coordinate system, *oxyz*, the axis of symmetry is axis *z*. The cross-section is shown in Figure 8b. The origin is used as the peak of the parabola, described in coordinates as:

(1) $$x^{2}+2bz=0$$

where *b* is the principal radius of the curvature of origin. Define the boundary value of parabolic axis (*x*, *z*) as *a* and *c*, respectively. Then, *b* can be expressed as *b* = *a*^2^/(2*c*), and *b* is twice the focal distance of parabola, *b* = 2*f*.

In Figure 8, the principal curvature is the direction of the meridian and circumference, respectively. The three axis are (*φ*, *θ*, *v*), where *φ* is the angle between the normal vector of the tangent and the axis of symmetry on the bell-shaped resonator; *θ* is the circumference angle; *v* is the thickness of the resonator. The variable basic relationship is as follows in Appendix A.

In the orthogonal curvilinear coordinate system, it is not only convenient to describe the point motion, but also to express the thickness of the resonator. The vibratory character of the bell-shaped resonator is presented in this coordinate.

### 3.2. Shell Equations of the Resonator

Based on the theory of thin shells and the Kirchhoff-Lyav hypothesis, the bell-shaped resonator's character as presented includes the relationship of internal force, the equilibrium differential equation and the dynamic equation [1,34,35].

#### 3.2.1. Strain Equation

For the isotropic three-dimensional elastic shell element, the middle surface geometric equation of the bell-shaped resonator is obtained in the orthogonal curvilinear coordinate system, (*φ*, *θ*, *v*). It can be expressed as:

(2) $$\{\begin{array}{l} {ɛ}_{\varphi }=\frac{1}{A_{\varphi }}\frac{\partial u}{\partial \varphi }+\frac{1}{A_{\varphi }A_{\theta }}\frac{\partial A_{\varphi }}{\partial \theta }v+k_{\varphi }w\\ {ɛ}_{\theta }=\frac{1}{A_{\varphi }}\frac{\partial v}{\partial \theta }+\frac{1}{A_{\varphi }A_{\theta }}\frac{\partial A_{\theta }}{\partial \varphi }u+k_{\theta }w\\ {ɛ}_{\varphi \theta }=\frac{A_{\varphi }}{A_{\theta }}\frac{\partial }{\partial \theta }\left(\frac{u}{A_{\varphi }}\right)+\frac{A_{\theta }}{A_{\varphi }}\frac{\partial }{\partial \varphi }\left(\frac{v}{A_{\theta }}\right)\\ \chi_{\varphi }=\frac{\partial k_{\varphi }}{\partial \varphi }\frac{u}{A_{\varphi }}+\frac{\partial k_{\varphi }}{\partial \theta }\frac{v}{A_{\theta }}-k_{\varphi }^{2}w-\frac{1}{A_{\varphi }}\frac{\partial }{\partial \alpha }\left(\frac{1}{A_{\varphi }}\frac{\partial w}{\partial \varphi }\right)-\frac{1}{A_{\varphi }A_{\theta }^{2}}\frac{\partial A_{\varphi }}{\partial \theta }\frac{\partial w}{\partial \theta }\\ \chi_{\theta }=\frac{\partial k_{\theta }}{\partial \theta }\frac{v}{A_{\theta }}+\frac{\partial k_{\varphi }}{\partial \theta }\frac{u}{A_{\varphi }}-k_{\theta }^{2}w-\frac{1}{A_{\theta }}\frac{\partial }{\partial \theta }\left(\frac{1}{A_{\theta }}\frac{\partial w}{\partial \theta }\right)-\frac{1}{A_{\varphi }^{2}A_{\theta }}\frac{\partial A_{\theta }}{\partial \varphi }\frac{\partial w}{\partial \varphi }\\ X_{\varphi \theta }=\frac{k_{\varphi }-k_{\theta }}{2}\left[\frac{A_{\varphi }}{A_{\theta }}\frac{\partial }{\partial \theta }\left(\frac{u}{A}\right)-\frac{A_{\theta }}{A_{\varphi }}\frac{\partial }{\partial \varphi }\left(\frac{v}{A_{\theta }}\right)\right]-\frac{1}{A_{\varphi }A_{\theta }}\left(\frac{\partial^{2}w}{\partial \varphi \partial \theta }-\frac{1}{A_{\varphi }}\frac{\partial A_{\varphi }}{\partial A_{\theta }}\frac{\partial w}{\partial \varphi }-\frac{1}{A_{\theta }}\frac{\partial A_{\theta }}{\partial_{\varphi }}\frac{\partial w}{\partial \theta }\right) \end{array}$$

where, *u*, *v*, *w* is the normal distance of three axes; *ε_φ_*, *ε_θ_* is linear strain of the direction, *φ* and *θ*, respectively; *ε_φθ_* is shearing strain between *φ* and *θ*; *χ_φ_*, *χ_θ_* is the change of principal curvature, *k_φ_* and *k_θ_*, of the point; *χ_φθ_* is the change of torsion, *φ* and *θ*. The variables are all on the middle surface. When we calculate them, the linear strain on the bell-shaped resonator is expressed as:

(3) $$\left\{ \begin{array}{l} e_{\varphi }={ɛ}_{\varphi }+v\chi_{\varphi }\\ e_{\theta }={ɛ}_{\theta }+v\chi_{\theta }\\ e_{\varphi \theta }={ɛ}_{\varphi \theta }+2v\chi_{\varphi \theta } \end{array}\right.$$

Substituting Equation (2) into Equation (3), the linear strain is as follows:

(4) $$e_{\varphi }\left[\frac{\cos^{3}\varphi }{b}\left(\frac{\partial u}{\partial \varphi }+\omega \right)\right]+v\left[\frac{\cos^{6}\varphi }{b^{2}}\left(\frac{\partial u}{\partial \varphi }-\frac{\partial^{2}w}{\partial \varphi^{2}}\right)-\frac{3\cos^{5}\varphi \sin\varphi }{b^{2}}\left(u-\frac{\partial w}{\partial \varphi }\right)\right]$$

(5) $$e_{\theta }=\left[\frac{\cos\varphi }{b\sin\varphi }\left(\frac{\partial v}{\partial \theta }+u\cos\varphi +w\sin\varphi \right)\right]+v\left[\frac{\cos^{2}\varphi }{b^{2}\sin\varphi }\left(\frac{\partial v}{\partial \theta }+\frac{1}{\sin\varphi }\frac{\partial^{2}w}{\partial \theta^{2}}+u\cos^{3}\varphi +\cos^{3}\varphi \frac{\partial w}{\partial \varphi }\right)\right]$$

(6) $$e_{\varphi \theta }=\left[\frac{\cos\varphi }{b\sin\varphi }\left(\frac{\partial u}{\partial \theta }+\cos^{2}\varphi \sin\varphi \frac{\partial v}{\partial \varphi }-v\cos\varphi \right)\right]+v[\frac{\cos^{4}\varphi }{b^{2}\sin\varphi }\frac{\partial u}{\partial \theta }-\frac{2\cos^{4}\varphi }{b^{2}\sin\varphi }\frac{\partial^{2}w}{\partial \theta \partial \varphi }+\frac{\cos^{4}\varphi }{b^{2}}\frac{\partial v}{\partial \varphi }+\frac{2\cos^{3}\varphi }{b^{2}\sin^{2}\varphi }\frac{\partial w}{\partial \theta }+(\frac{\cos^{3}\varphi -\cos^{3}\varphi }{b^{2}\sin\varphi })v]$$

#### 3.2.2. Internal Force

Based on the transformation relation in the orthogonal curvilinear coordinate system and the expression of internal force in the theory of thin shells, the internal force of the bell-shaped resonator is presented. Using the relations between the mechanical force and stress, one can derive the mechanical membrane forces and bending moments for the bell-shaped resonator [32–34]:

(7) $$F_{T\varphi }=\frac{Eh(\varphi )}{1-\mu^{2}}\left({ɛ}_{\varphi }+\mu {ɛ}_{\theta }\right)=\frac{Eh(\varphi )}{1-\mu^{2}}\left[\frac{\cos\varphi }{b\sin\varphi }\left(\frac{\partial v}{\partial \theta }+u\cos\varphi +w\sin\varphi \right)+\mu \frac{\cos^{3}\varphi }{b}\left(\frac{\partial u}{\partial \varphi }+w\right)\right]$$

(8) $$F_{T\theta }=\frac{Eh(\varphi )}{1+\mu^{2}}\left({ɛ}_{\theta }+\mu {ɛ}_{\varphi }\right)=\frac{Eh(\varphi )}{\left(1+\mu^{2}\right)}\left[\frac{\cos^{3}\varphi }{b}\left(\frac{\partial u}{\partial \varphi }+w\right)+\mu \frac{\cos\varphi }{b\sin\varphi }\left(\frac{\partial v}{\partial \theta }+u\cos\varphi +w\sin\varphi \right)\right]$$

(9) $$F_{T\varphi \theta }=\frac{Eh(\varphi )}{2\left(1+\mu \right)}{ɛ}_{\varphi \theta }=\frac{Eh(\varphi )}{2\left(1+\mu \right)}\left[\frac{\cos\varphi }{b\sin\varphi }\left(\frac{\partial u}{\partial \theta }+\cos^{2}\varphi \sin\varphi \frac{\partial v}{\partial \varphi }-v\cos\varphi \right)\right]$$

(10) $$M_{\varphi }=D\left(\chi_{\varphi }+\mu \chi_{\theta }\right)=D\{\left[\frac{\cos^{2}\varphi }{b^{2}\sin\varphi }\left(\frac{\partial u}{\partial \theta }-\frac{1}{\sin\varphi }\frac{\partial^{2}w}{\partial \theta^{2}}+u\cos^{3}\varphi -\cos^{3}\varphi \frac{\partial w}{\partial \varphi }\right)\right]+\mu \left[\frac{\cos^{6}\varphi }{b^{2}}\left(\frac{\partial u}{\partial \varphi }-\frac{\partial^{2}w}{\partial \varphi^{2}}\right)-\frac{3\cos^{5}\varphi \sin\varphi }{b^{2}}\left(u-\frac{\partial w}{\partial \varphi }\right)\right]\}$$

(11) $$M_{\theta }=D\left(\chi_{\theta }+\mu \chi_{\varphi }\right)=D\{\left[\frac{\cos^{6}\varphi }{b^{2}}\left(\frac{\partial u}{\partial \varphi }-\frac{\partial^{2}w}{\partial \varphi^{2}}\right)-\frac{3\cos^{5}\varphi \sin\varphi }{b^{2}}\left(u-\frac{\partial w}{\partial \varphi }\right)\right]+\mu \left[\frac{\cos^{2}\varphi }{b^{2}\sin\varphi }\left(\frac{\partial u}{\partial \varphi }-\frac{1}{\sin\varphi }\frac{\partial^{2}w}{\partial \theta^{2}}+u\cos^{3}\varphi -\cos^{3}\varphi \frac{\partial w}{\partial \varphi }\right)\right]\}$$

(12) $$M_{\varphi \theta }=M_{\theta \varphi }=\left(1-\mu \right)D\chi_{\varphi \theta }=\left(1-\mu \right)D[\frac{\cos^{4}\varphi }{b^{2}\sin\varphi }\frac{\partial u}{\partial \theta }-\frac{2\cos^{4}\varphi }{b^{2}\sin\varphi }\frac{\partial w}{\partial \theta \partial \varphi }+\frac{\cos^{4}\varphi }{b^{2}}\frac{\partial v}{\partial \varphi }+\frac{2\cos^{3}\varphi }{b^{2}\sin^{2}\varphi }\frac{\partial w}{\partial \theta }+(\frac{\cos^{5}\varphi -2\cos^{3}\varphi }{2b^{2}\sin\varphi })v]$$

where $D=\frac{Eh{\left(\varphi \right)}^{3}}{12(1-\mu^{2})}$ is the flexural rigidity of the thin shell; dimension: *L*^2^*MT*^−2^; *μ* is the Poisson ratio and *E* is the Young modulus. The bell-shaped resonator is of equal thickness, so *h* (*φ*) = *h*.

#### 3.2.3. Equilibrium Differential Equation

According the the D'Alembert principle, if the sum of force and inertial force and the torque are all equal to zero, which is applied to the isolated unit, then the unit is in a state of equilibrium. The bell-shaped resonator is fit for this principle. As far as we know, the middle surface of the bell-shaped resonator cannot stretch. The three components of tangential displacement in the shell bending equation are equal to zero. It can be expressed as [1]:

(13) $${ɛ}_{\varphi }={ɛ}_{\theta }={ɛ}_{\varphi \theta }=0$$

The normal force and shearing force are equal to zero:

(14) $$F_{T\varphi }=F_{T\theta }=F_{T\varphi \theta }=0$$

Therefore, we can derive the equilibrium differential equation (for specific derivation, see Appendix B):

(15) $$\{\begin{array}{l} \frac{\partial M_{\varphi \theta }}{\partial \theta }-\cos\varphi M_{\theta }+\cos\varphi M_{\varphi }+\cos^{2}\varphi \sin\varphi \frac{\partial M_{\varphi }}{\partial \varphi }=-\frac{b^{2}\sin\varphi }{\cos^{4}\varphi }q_{\varphi }\\ \frac{2}{\cos\varphi }M_{\varphi \theta }+\sin\varphi \frac{\partial M_{\varphi \theta }}{\partial \varphi }+\frac{1}{\cos^{2}\varphi }\frac{\partial M_{\theta }}{\partial \theta }=-\frac{b^{2}\sin\varphi }{\cos^{4}\varphi }q_{\theta }\\ \sin\varphi M_{\theta }-\sin\varphi M_{\varphi }+\cos\varphi \left(2-3\sin^{2}\varphi \right)\frac{\partial M_{\varphi }}{\partial \varphi }-\cos\varphi \frac{\partial M_{\theta }}{\partial \varphi }\\ +\frac{2}{\cos_{\varphi }\sin_{\varphi }}\frac{\partial M_{\varphi \theta }}{\partial \theta }+\cos^{2}\varphi \sin\varphi \frac{\partial^{2}M_{\varphi }}{\partial \varphi^{2}}+\frac{1}{\sin\varphi \cos^{2}\varphi }\frac{\partial^{2}M_{\theta }}{\partial \theta^{2}}+2\frac{\partial^{2}M_{\varphi \theta }}{\partial \theta \partial \varphi }=-\frac{b^{2}\sin\varphi }{\cos^{4}\varphi }qv \end{array}$$

where *q_φ_*, *q_θ_*, *q*_v_ is the middle surface load of the bell-shaped resonator, which is in relation to the input angular velocity. For the point, *P* (*φ*, *θ*, *v*), on the bell-shaped resonator, the motion expression in the vector is expressed as:

(16) $$\varphi =\varphi \hat{\varphi },\theta =\theta \hat{\theta },\text{v}=v\hat{v}$$

where ***φ̂***, ***θ̂***, ***v̂*** is the unit vector of the coordinate axis. The motion vector, ***R***, of the point, *P*, is as follows:

(17) $$\text{R}=u\hat{\varphi }+v\hat{\theta }+w\hat{v}$$

The angular velocity, **Ω**, is along the axis of symmetry in the inertial space; the expression is:

(18) $$\Omega =\Omega \hat{k}=\Omega (\hat{v}\cos\varphi -\hat{\varphi }\sin\varphi )$$

where ***k̂*** = ***v̂*** cos *φ* – ***φ^*** sin *φ* is the unit vector on axis *z* [10].

According to the Coriolis Theorem, the absolute velocity of point *P* motion relative to inertial space can be expressed as:

(19) $$\text{V}=\frac{d\text{R}}{dt}+\Omega \times \text{R}=(\dot{u}-v\Omega \cos\varphi )\hat{\varphi }+(\dot{v}+v\Omega \cos\varphi +w\Omega \sin\varphi )\hat{\theta }+(\dot{w}-v\Omega \sin\varphi )\hat{v}$$

The absolute acceleration is as follows:

(20) $$\text{a}=\frac{d\text{V}}{dt}=(\ddot{u}-\dot{v}\Omega \cos\varphi )\hat{\varphi }+(\ddot{v}+\dot{v}\Omega \cos\varphi +\dot{w}\Omega \sin\varphi )\hat{\theta }+(\ddot{w}-\dot{v}\Omega \sin\varphi )\hat{v}$$

The load of rotation is the load of the middle surface on the bell-shaped resonator and derived in:

(21) $$\{\begin{array}{l} q_{\varphi }=ph(\ddot{u}-\dot{v}\Omega \cos\varphi )\\ q_{\theta }=ph(\ddot{v}+\dot{v}\Omega \cos\varphi +\dot{w}\Omega \sin\varphi )\\ qv=ph(\ddot{w}-\dot{v}\Omega \sin\varphi ) \end{array}$$

Substituting Equation (15) into Equation (21) derives the equilibrium differential equation:

(22) $$\{\begin{array}{l} \frac{\partial M_{\varphi \theta }}{\partial \theta }-\cos\varphi M_{\theta }+\cos\varphi M_{\varphi }+\cos^{2}\varphi \sin\varphi \frac{\partial M_{\varphi }}{\partial \varphi }=-\frac{b^{2}\sin\varphi }{\cos^{4}\varphi }ph(\ddot{u}-\dot{v}\Omega \cos\varphi )\\ \frac{2}{\cos\varphi }M_{\varphi \theta }+\sin\varphi \frac{\partial M_{\varphi \theta }}{\partial \varphi }+\frac{1}{\cos^{2}\varphi }\frac{\partial M_{\theta }}{\partial \theta }=-\frac{b^{2}\sin\varphi }{\cos^{4}\varphi }ph(\ddot{v}+\dot{v}\Omega \cos\varphi +\dot{w}\Omega \sin\varphi )\\ \sin\varphi M_{\theta }-\sin\varphi M_{\varphi }+\cos\varphi (2-3\sin^{2}\varphi )\frac{\partial M_{\varphi }}{\partial \varphi }-\cos\varphi \frac{\partial M_{\theta }}{\partial \varphi }+\frac{2}{\cos\varphi \sin\varphi }\frac{\partial M_{\varphi \theta }}{\partial \theta }\\ +\cos^{2}\varphi \sin\varphi \frac{\partial^{2}M_{\varphi }}{\partial \varphi^{2}}+\frac{1}{\sin\varphi \cos^{2}\varphi }\frac{\partial^{2}M_{\theta }}{\partial \theta^{2}}+2\frac{\partial^{2}M_{\varphi \theta }}{\partial \theta \partial_{\varphi }}=-\frac{b^{2}\sin\varphi }{\cos^{4}\varphi }ph(\ddot{w}-\dot{v}\Omega \sin\varphi ) \end{array}$$

### 3.3. The Second Order Normal Vibration Mode Dynamical Equation

To solve the Equation (15), the displacement vector of an arbitrary point in the bell-shaped resonator is developed according to the second order normal vibration mode, which is not stretched:

(23) $$\begin{array}{c} u(\varphi ,\theta ,t)=U(\varphi )[p(t)\cos(2\theta )+q(t)\sin(2\theta )]\\ v(\varphi ,\theta ,t)=V(\varphi )[p(t)\sin(2\theta )-q(t)\cos(2\theta )]\\ w(\varphi ,\theta ,t)=W(\varphi )[p(t)\cos(2\theta )+q(t)\sin(2\theta )] \end{array}$$

where *U* (*φ*), *V* (*φ*), *W* (*φ*) is the Rayleigh functions about the bell-shaped resonator's second order intrinsic vibration mode on the three axes, respectively. *p* (*t*), *q* (*t*) is the displacement of the vibratory rigid axis.

According to Equations (13) and (14), the relationship is:

(24) $$\{\begin{array}{l} \frac{\partial u}{\partial \varphi }+w=0\\ \frac{\cos\varphi }{b\sin\varphi }\left(\frac{\partial v}{\partial \theta }+\cos\varphi u+\sin\varphi w\right)=0\\ \frac{\cos\varphi }{b\sin\varphi }\left(\frac{\partial u}{\partial \theta }+\frac{\partial v}{\partial \theta }\cos^{2}\varphi \sin\varphi -v\cos\varphi \right)=0 \end{array}$$

Equation (24) describes the boundary condition of the bell-shaped resonator. The solution of this equation is named the mode shape function or Rayleigh functions [33]:

(25) $$\{\begin{array}{lll} U(\varphi ,\theta ) & = & A_{n}\cos\left[(2n+1)\pi \varphi /\varphi^{b}\right]\sin^{n+1}\varphi \cos(n\theta )\\ V(\varphi ,\theta ) & = & -A_{n}\cos\varphi \sin^{n+1}\varphi \sin(n\theta )\\ W(\varphi ,\theta ) & = & A_{n}(n+1)\cos\varphi \sin^{n}\varphi \cos(n\theta ) \end{array}$$

where *A_n_* is the coefficient of the vibratory amplitude; *n* is the order of the resonator vibration. According to the Equation (25), the Rayleigh functions about the bell-shaped resonator's second order normal vibration mode are:

(26) $$\{\begin{array}{lll} U(\varphi ) & = & A_{n}\cos\left[5\pi \varphi /\varphi^{b}\right]\sin^{3}\varphi \\ V(\varphi ) & = & -A_{n}\cos\varphi \sin^{3}\varphi \\ W(\varphi ) & = & A_{n}3\cos\varphi \sin^{2}\varphi \end{array}$$

Substituting Equation (23) into Equation (22), the differential equation is about *p* (*t*) and *q* (*t*). Calculating using Maple finds that the equation is too complex to solve using the traditional method. For the HRG, the equation is easier than the BVG, which is solved through the Bubnov-Galerkin method [36-38]. The equation of BVG is too complex to simplify (for specific derivation, see Appendix C).

For Equation (22), the equilibrium differential equation and dynamic equation are presented, which include control force and torque. It means that the governing equation of the control loop is achieved about the bell-shaped resonator. For the Coriolis Vibratory Gyro, the simplified second order normal mode of the resonator is the second order orthogonal spring system. It can be expressed as [1,10,39,40]:

(27) $$\{\begin{array}{c} m_{0}\ddot{p}-2\Omega b\dot{q}+c_{0}p+\beta q=f_{p}\\ m_{0}\ddot{q}+2\Omega b\dot{p}+c_{0}q+\beta p=f_{q} \end{array}$$

where *m*_0_, *b*, *c*_0_, *β* is an undetermined function about *φ*, *θ*, *h* and other material parameters. Therefore, the natural frequency of the bell-shaped resonator is:

(28) $$\omega_{n}=\sqrt{\frac{c_{0}}{m_{0}}}$$

The precession factor is:

(29) $$K=\frac{b}{2m_{0}}$$

The typical analytical method does not solve this complex problem, but it clearly describes the vibratory procedure, the force and equilibrium relation. In practice, the Finite Element Method is widely used for the bell-shaped resonator to analyze the natural frequency, mode shape and work mode.

### 3.4. Mode Shape of Resonator

**Figure 9.**
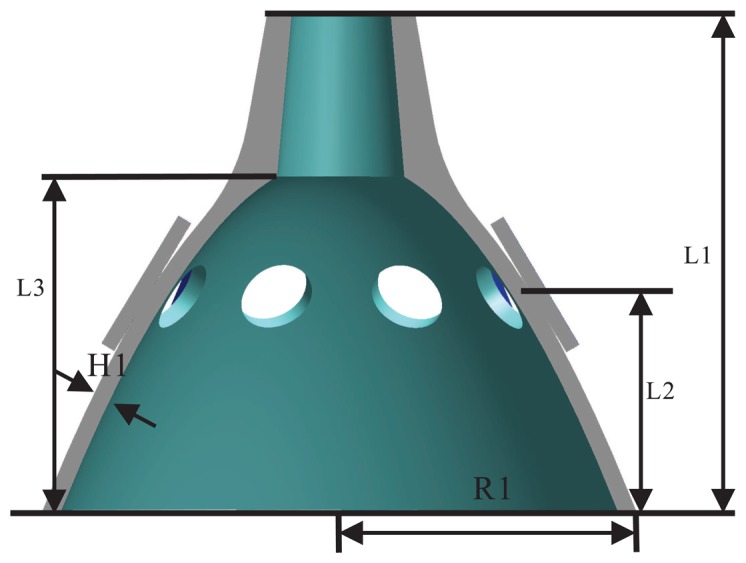
Structure parameters of the bell-shaped resonator.

**Figure 10.**
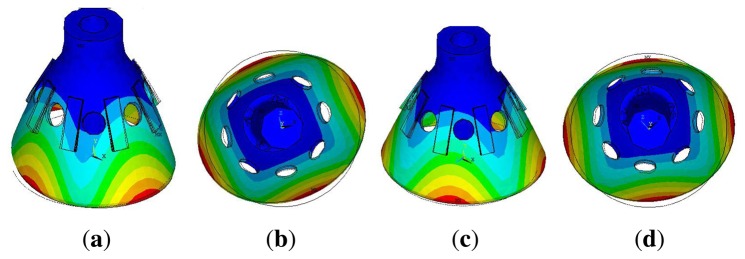
Schematic of the work mode shape. (**a**) Front View of Primary Mode. (**b**) Bottom View of Primary Mode. (**c**) Front View of Second Mode. (**d**) Bottom View of Second Mode.

Analysis of the vibratory character is based on the theory of thin shells. It is very necessary to know the overall mode shape of the bell-shaped resonator. The modal simulation is presented using FEM. The main structure parameters of the resonator are as follows: *L*1 = 21 mm, *R*1 = 11 mm, *H*1 = 0.7 mm, *L*2 = 9 mm, *L*3 = 15 mm; as shown in Figure 9. The material parameters and simulation condition is followed in Table 1. The modal analyzed results show the work mode shape in Figure 10, and the natural frequency is: 5, 909.3 Hz. The mode shape is a four antinode shape.

**Figure 11.**
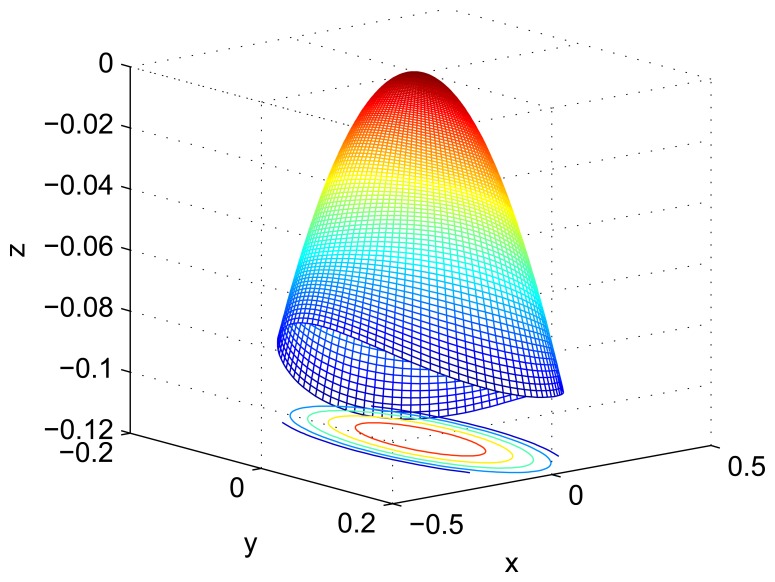
Numerical simulation of the mode shape.

Using the mode shape function of Section 3.3, the simulation result is shown in Figure 11 in Matlab. This demonstrates that the Equation (25) is similar as the result of FEM. However, the amplitude of the mode shape is different between them. The reason is about the error of simulation and amplitude coefficient.

## 4. Governing Equation and Signal Process

**Figure 12.**
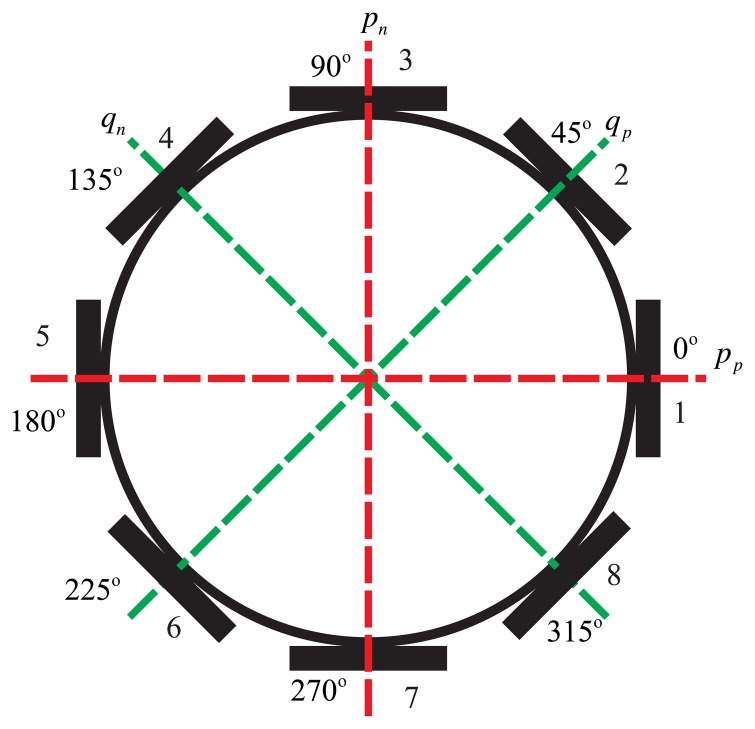
The transversal surface of the standing wave.

The mode shape control about the standing wave on the circumference is very important for the BVG. Equation (27) describes the second order normal mode of the resonator that is a traditional second order spring system. This type of equation is a simplified Foucault Pendulum function. The form of the standing wave is the foundation of the governing equation and signal process of the BVG. The transversal surface of the standing wave of the bell-shaped resonator is shown in Figure 12.

The transversal surface of the standing wave is the vibratory ring. On the ring, there are two rigid axes, *p* and *q*. *p* is the axis of the primary mode, and *q* is the axis of the second mode [41,42]. Coriolis force couples the motion between two axes, *p* and *q*. In Figure 12, the *p_n_* is the control axis of *p* (first and fifth element); *p_p_* is the sensitive axis of *p* (third and seventh element); *q_n_* is the control axis of *q* (second and sixth element); *q_p_* is the sensitive axis of *q* (fourth and eighth element). The relationship of *p* and *q* is described in Equation (27). In the literature [15,16,36,39], researchers present the general vibratory equation for the coriolis vibratory gyro:

(30) $$\{\begin{array}{c} \ddot{p}-2nK\Omega \dot{q}+\left(\frac{2}{\tau }+\Delta \left(\frac{1}{\tau }\right)\cos2n\theta_{\tau }\right)\dot{p}+\Delta \left(\frac{1}{\tau }\right)\sin2n\theta_{\tau }\dot{q}\\ +(\omega^{2}-\omega \Delta \omega \cos2n\theta_{\omega })p-\omega \Delta \omega \sin2n\theta_{\omega }q=f_{p}\\ \ddot{q}+2nK\Omega \dot{p}+\left(\frac{2}{\tau }-\Delta \left(\frac{1}{\tau }\right)\cos2n\theta_{\tau }\right)\dot{q}+\Delta \left(\frac{1}{\tau }\right)\sin2n\theta_{\tau }\dot{p}\\ +(\omega^{2}+\omega \Delta \omega \cos2n\theta_{\omega })q-\omega \Delta \omega \sin2n\theta_{\omega }p=f_{q} \end{array}$$

where *K* is the precession factor; *n* is the mode order of the resonator; *f_p_* is the force applied to *p*; *f_q_* is the force applied to *q*; Ω is the angular velocity; *ω_p_* is the frequency of the primary mode; *ω_q_* is the frequency of the secondary mode; *θ_ω_* is the angle between the primary axis and *p*; *θ_τ_* is the angle between the damping axis and *p*; *τ_p_*, *τ_q_* is the delay time of the axis; $\omega^{2}=\frac{\omega_{p}^{2}+\omega_{q}^{2}}{2}$, ${\Delta \omega }^{2}=\frac{\omega_{p}^{2}-\omega_{q}^{2}}{2}$, $\frac{1}{\tau }=\frac{1}{2}\left(\frac{1}{\tau_{p}}+\frac{1}{\tau_{q}}\right)$, $\Delta \left(\frac{1}{\tau }\right)=\left(\frac{1}{\tau_{p}}-\frac{1}{\tau_{q}}\right)$.

**Figure 13.**
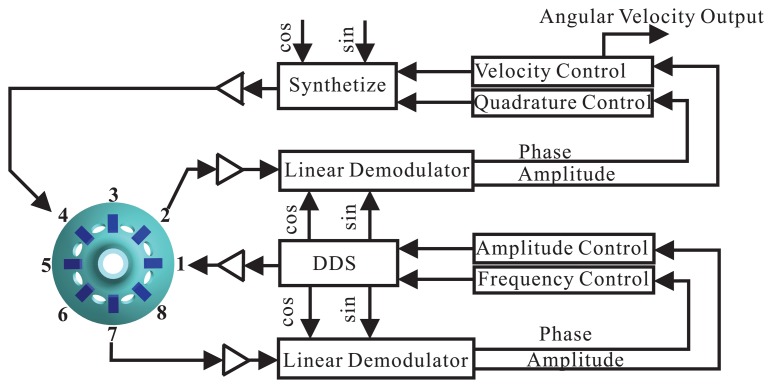
Schematic of the signal process.

The diagram of the BVG signal process method is shown in Figure 13. The control loops include an amplitude control loop, a frequency control loop, a quadrature control loop and a rate control loop [13–15,18]. The authors had written an article about this in the The 32nd Chinese Control Conference [40]. It will be published in July 2013.

## 5. Analysis of Impact Dynamic

**Figure 14.**
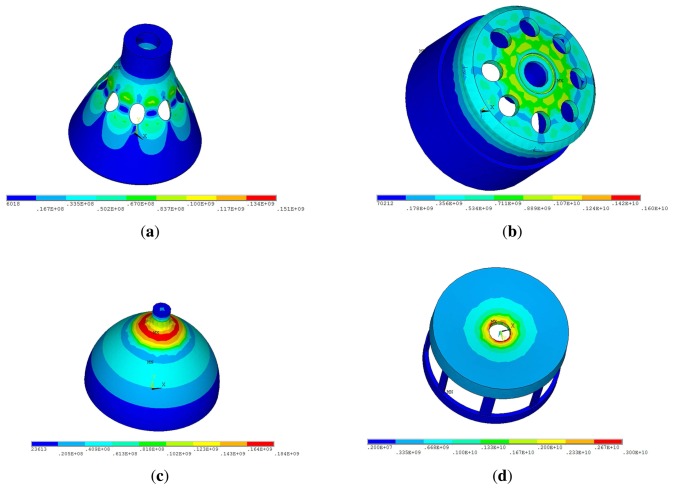
The stress cloud charts under 20,000 × g. (**a**) Stress Cloud Charts of BVG. (**b**) Stress Cloud Charts of Cylinder Vibratory Gyro. (**c**) Stress Cloud Charts of Hemispherical Resonator Gyro. (**d**) Stress Cloud Charts of Novel Ring Vibratory Gyro.

The bell-shaped vibratory angular rate gyro's greatest strength is its remarkable impact resistance property. Using FEM to compare the hemispherical resonator gyro [1], the cylinder vibratory gyro [17] and the novel ring vibratory gyro [18] in the 20,000 × g gravitational field, the stress cloud charts are shown in Figure 14. All the gyro have the same height (21 mm), the same edge width (22 mm) and the same thickness (0.7 mm) using Ni43CrTi. The simulation condition is the same same as the paper [24].

The maximum stress of HRG is 181 MPa; the cylinder vibratory gyro is 1,600 MPa; the novel ring vibratory gyro is 3,000 MPa; the BVG is 151 MPa. The result shows that the bell-shaped resonator has the best impact resistance property. Based on the mechanics of materials, the tensile strength of Ni43CrTi is 500 MPa. Therefore, the bell-shaped resonator does not get damaged in 20,000 × g. During the impact process, the resonator produces a small deformation. It is quickly restored after the impact. The control loop also can restrain the influence of the small deformation, too.

## 6. Fabrication and Experiments

### 6.1. Fabrication

**Figure 15.**
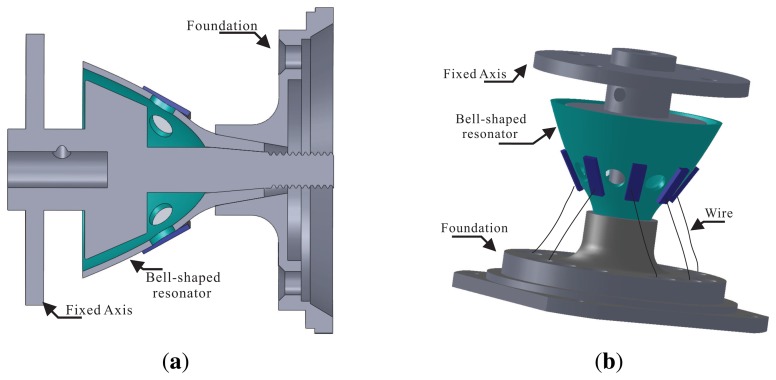
Schematic of fabrication. (**a**) Cross-Section of fabrication. (**b**) Total View.

The main structure of the BVG includes the bell-shaped resonator, a fixed axis and a foundation. These connect together through mechanical technology, as shown in Figure 15. The procedure is as follows:

**Step1:** The piezoelectric element with the wire attached on the wall of the bell-shaped resonator;
**Step2:** The fixed axis fastens the bell-shaped resonator to the foundation;
**Step3:** The wire connects to the insulated joint on the foundation.

**Figure 16.**
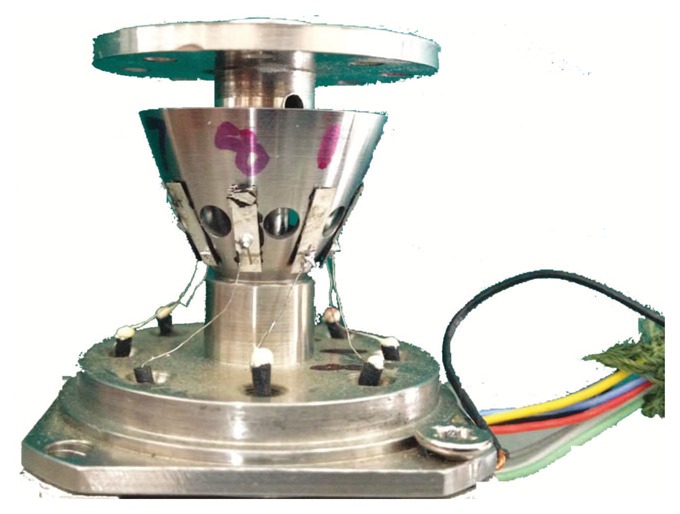
Prototypal bell-shaped resonator.

Finally, the 9 wires connected to the signal process system include 8 wires of piezoelectric elements and GND. The wires connect to the circuit board through the insulated joint, which is installed on the foundation. The prototypal bell-shaped resonator is shown in Figure 16.

### 6.2. Experiment

The experiment of the bell-shaped resonator includes: natural frequency test, mode shape test, coriolis test and gyroscopic effect test. The natural frequency and mode shape have been tested in [24]. In this paper, it focuses on the other test.

#### 6.2.1. Coriolis Test

**Figure 17.**
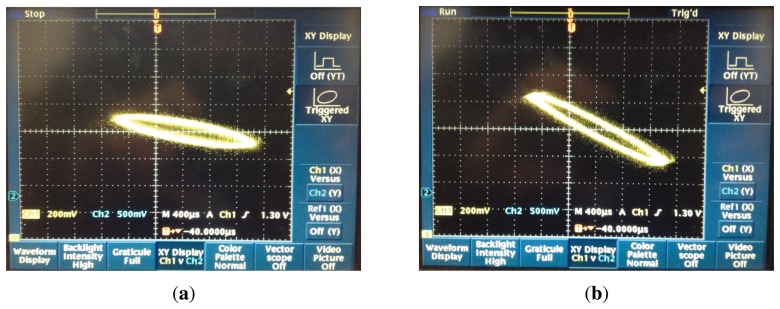
Picture of coriolis test. (**a**) Ω = 0. (**b**) Ω ≠ 0.

The sine alternating current signal (*V_pp_* = 10V) is applied to the first and fifth element. In order to readout the signal clearly, the output signal of the piezoelectric element is processed by the operator amplitude and summed by the piezoelectric element with the same axis. In the test, the Lissajous pattern is a good method to analyze the coriolis effect about the two axes, *p* and *q*. The oscilloscope that was chosen is the Tektronix TDS 3032B. Channel 1 connects to the *p_p_* axis, used as x. Channel 2 connects the *q_p_* axis, used as y. When Ω = 0, the picture is as shown in Figure 17a. When Ω ≠ 0, the picture is as shown in Figure 17b.

During the test, the bell-shaped resonator is in an uncontrolled situation. The frequency split of the tested bell-shaped resonator is 0.5 Hz. In the static state, the two axes exit the vibratory coupling. This described Lissajous pattern is not the line. When the angular velocity is applied to the sensitive axis, the couple effect is very obvious and the standing wave produces precession. The bell-shaped resonator is already sensitive to the coriolis effect.

#### 6.2.2. Gyroscopic Effect Test

**Figure 18.**
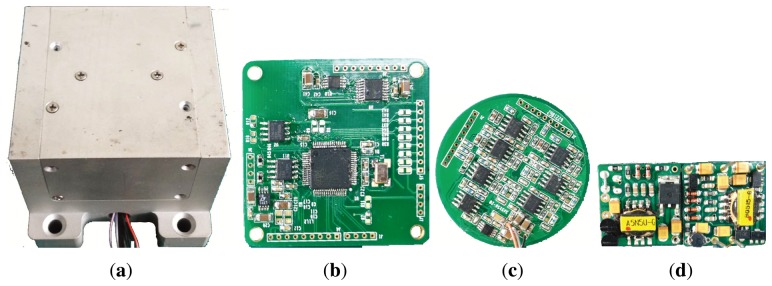
The photo of circuit. (**a**) Gyro chassis; (**b**) signal processing; (**c**) signal sample; (**d**) Power.

The prototypal BVG includes power, a signal sample board and a signal processing board, as shown in Figure 18.

**Figure 19.**
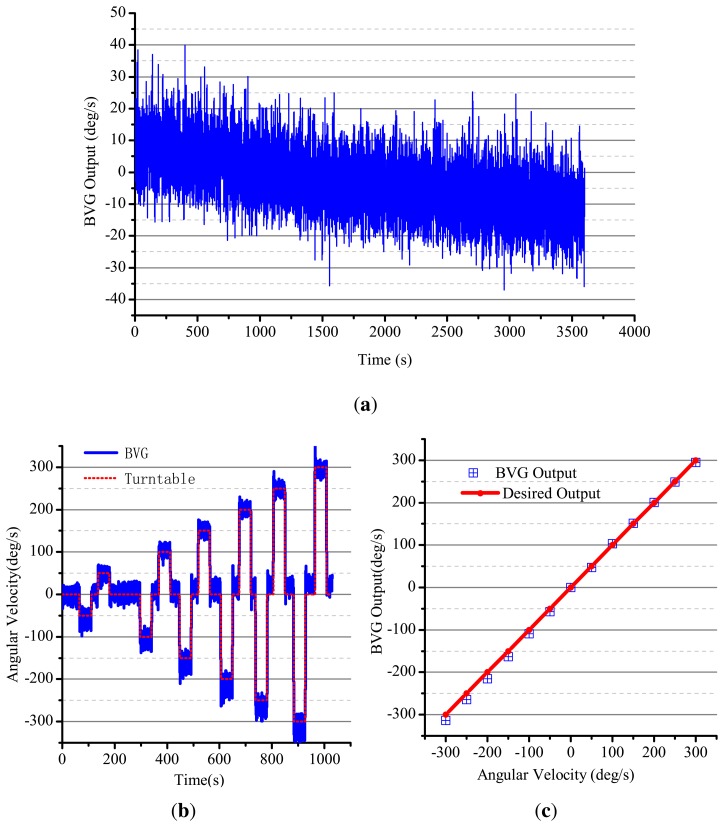
Experiment result. (**a**) Drift curve; (**b**) Range curve; (**c**) Linearity.

The BVG is fixed on the high-precision angular velocity turntable to test the gyroscopic effect. The results are shown in Figure 19. According to the original output of the BVG, only thick calibration of the BVG is convenient for analysis. The range of the BVG is about ±300°/s. The zero drift curve is shown in Figure 19a. The noise of the gyro is very large, and the signal to noise ratio (SNR) is low. The dynamic experiment curve is shown in Figure 19b,c. Using the 50 Hz FIRfilter, the zero drift instability is 22.5°/h and linearity is 1.24%. This index can be controlled by the filter. The index is poorer than the other shell vibratory gyro [13,14,18]. The reason for the poor performance is as follow:

- The manufacturing technology has imperfections. Specifically, the piezoelectric element is difficult to stick on the resonator with a curved surface of variable curvature.
- There is no temperature compensation for the bell-shaped resonator. The long work time leads to a change of the frequency and influences the drift. The bell-shaped resonators vibratory characteristics change as the temperature changes.
- Structure processing error and control loop error exist. Improving the method of restraining the frequency split and designing the control loop is needed.
- The parameters of the governing equation of the bell-shaped resonator are not exact.

In the future, important work is needed: improving the manufacturing technology and the control loop design, designing a temperature compensation method, *etc.* The performance of a prototypal BVG is not satisfactory, but the method of design and analysis is demonstrated perfect.

## 7. Conclusions

Based on the orthogonal curvilinear coordinate system, the equilibrium differential equation using the theory of thin shells is presented, and the motion of the point on the bell-shaped resonator is described. The vibratory character of the resonator is analyzed with the input angular velocity. On this basis, the mode shape function and the simplified second order normal vibration mode dynamical equation are obtained. The Coriolis coupling relationship about the primary mode and the secondary mode is established. The method of signal processing of the BVG and the control loop of the prototype is presented. To analyze the impact resistance property of the bell-shaped resonator, which is compared with the other shell resonator using the Finite Element Method, demonstrates that the BVG has the advantage of better impact resistance property. A reasonable means of installation and the prototypal gyro are designed. The gyroscopic effect of the BVG is characterized through experimentation. The experimental results shows that the zero drift instability is 22.5°/h and linearity is 1.24%. The index is poorer than other shell vibratory gyros. The main reason is: the manufacturing technology has imperfections; there is not any temperature compensation; structure processing error and control loop error exist; the parameters of the governing equation of the bell-shaped resonator are not exact. In the future, important work includes improvement of the performance of the BVG. In conclusion, the BVG has not only the advantages of low cost, low power, long work life, high sensitivity, and so on, but also a simple structure and better impact resistance for low and medium angular velocity measurements.

## Figures and Tables

**Table 1.** The parameters with simulation.

**Name of the Parameters (Unit)**	**Value**
Material	Ni43CrTi (3J53)
Density (*kg*/*m*^3^)	8,170
Poisson's ratio	0.3
Young modulus (*GPa*)	196.76
Yield strength (*MPa*)	500
Simulation method	Transient dynamic
Mesh generation method	Free
Release ratio	1*E* – 4
Piezoelectric element	PZT-5A

## A. Variable Basic Relationship

The variable basic relationship is as follows:

1. Differential of arc length:
  (31) $${\left(ds\right)}^{2}={R_{\varphi }}^{2}{\left(d\varphi \right)}^{2}+{R_{\theta }}^{2}\sin^{2}\varphi {\left(d\theta \right)}^{2}$$
2. Differential of volume:
  (32) $$dV=R_{\theta }\sin\varphi d\theta R_{\varphi }d\varphi dv$$
3. Curvature:
  (33) $$\begin{array}{ccc} k_{\varphi }= & \frac{1}{b{\left[1+{\left(\frac{x}{b}\right)}^{2}\right]}^{\frac{3}{2}}} & =\frac{\cos\varphi }{b}\\ k_{\theta }= & \frac{1}{b{\left[1+{\left(\frac{x}{b}\right)}^{2}\right]}^{\frac{1}{2}}} & =\frac{\cos^{3}\varphi }{b} \end{array}$$
4. Radius of curvature:
  (34) $$\begin{array}{c} R_{\varphi }=b{\left[1+{\left(\frac{x}{b}\right)}^{2}\right]}^{\frac{3}{2}}=\frac{b}{\cos\varphi }\\ R_{\theta }=b{\left[1+{\left(\frac{x}{b}\right)}^{2}\right]}^{\frac{1}{2}}=\frac{b}{\cos^{3}\varphi } \end{array}$$
5. Lame constants:
  (35) $$A_{\varphi }=R_{\varphi }=\frac{b}{\cos^{3}\varphi },A_{\theta }=R_{\theta }\sin\varphi =\frac{b\sin\varphi }{\cos\varphi }$$
6. Domain of definition:
  (36) $$\varphi_{t}\leq \varphi \leq \varphi_{b},0\leq \theta <2\pi ,-\frac{h(\varphi )}{2}\leq v\leq \frac{h(\varphi )}{2}$$
  where *φ_t_* is the angle between the normal vector of the tangent and the axis of symmetry on the bell-shaped resonator top edge; *φ_b_* is the bottom edge; *h* (*φ*) is the thickness of the resonator, which is about *φ.*

## B. Equilibrium Equation

According to the theory of thin shells, the basic equilibrium is as follows in Equation (37):

(37) $$\{\begin{array}{l} \frac{\partial }{\partial \alpha }(A_{2}F_{T1})-\frac{\partial A_{2}}{\partial \alpha }F_{T2}+\frac{\partial A_{1}}{\partial \beta }F_{T12}+\frac{\partial }{\partial \beta }(A_{1}F_{T12})+A_{1}A_{2}k_{1}F_{S1}+A_{1}A_{2}q_{1}=0\\ \frac{\partial }{\partial \beta }(A_{1}F_{T2})-\frac{\partial A_{1}}{\partial \beta }F_{T1}+\frac{\partial A_{2}}{\partial \alpha }F_{T12}+\frac{\partial }{\partial \alpha }(A_{2}F_{T12})+A_{1}A_{2}k_{2}F_{S2}+A_{1}A_{2}q_{2}=0\\ -A_{1}A_{2}(k_{1}F_{T1}+k_{2}F_{T2})+\frac{\partial }{\partial \alpha }(A_{2}F_{S1})+\frac{\partial }{\partial \beta }(A_{1}F_{S2})+A_{1}A_{2}q_{3}=0\\ \frac{\partial }{\partial \alpha }(A_{2}M_{12})+\frac{\partial A_{2}}{\partial \alpha }M_{12}-\frac{\partial A_{1}}{\partial \beta }M_{1}+\frac{\partial }{\partial \beta }(A_{1}M_{2})-A_{1}A_{2}F_{S2}=0\\ \frac{\partial }{\partial \beta }(A_{1}M_{12})+\frac{\partial A_{1}}{\partial \beta }M_{12}-\frac{\partial A_{2}}{\partial \alpha }M_{2}+\frac{\partial }{\partial \alpha }(A_{2}A_{1})-A_{1}A_{2}F_{S2}=0 \end{array}$$

For the orthogonal curvilinear coordinate system:

(38) $$\{\begin{array}{l} \frac{\partial }{\partial \varphi }(A_{2}F_{T\varphi })-\frac{\partial A_{2}}{\partial \varphi }F_{T\theta }+\frac{\partial A_{1}}{\partial \theta }F_{T\varphi \theta }+\frac{\partial }{\partial \theta }(A_{1}F_{T\varphi \theta })+k_{1}Q_{1}+A_{1}A_{2}q_{1}=0\\ \frac{\partial }{\partial \theta }(A_{1}F_{T\theta })-\frac{\partial A_{1}}{\partial \theta }F_{T\varphi }+\frac{\partial A_{2}}{\partial \varphi }F_{T\varphi \theta }+\frac{\partial }{\varphi }(A_{2}F_{T\varphi \theta })+k_{2}Q_{2}+A_{1}A_{2}q_{2}=0\\ -A_{1}A_{2}(k_{1}F_{T\varphi }+k_{2}F_{T\theta })+\frac{\partial }{\partial \varphi }\left(\frac{Q_{1}}{A_{1}}\right)+\frac{\partial }{\partial \theta }\left(\frac{Q_{2}}{A_{2}}\right)+A_{1}A_{2}q_{3}=0 \end{array}$$

Where $Q_{1}=\frac{\partial }{\partial \theta }(A_{1}M_{\varphi \theta })+\frac{\partial A_{1}}{\partial \theta }M_{\varphi \theta }-\frac{\partial A_{2}}{\partial \varphi }M_{\theta }+\frac{\partial }{\partial \varphi }(A_{2}M_{\varphi })$, $Q_{2}=\frac{\partial }{\partial \varphi }(A_{2}M_{\varphi \theta })+\frac{\partial A_{2}}{\partial \varphi }M_{\varphi \theta }-\frac{\partial A_{1}}{\partial \theta }M\varphi +\frac{\partial }{\partial \theta }(A_{1}M\theta )$.

Using the hypothesis condition in the paper, the equilibrium differential equation is derived.

## C. Solution of Equilibrium Equation

The solution of Equation (22) is derived by the Bubnov-Galerkin method [1,34,38,39,43]. Substituting Equations 10–12 into Equation (15), as follows in Equation (39):

(39) $$\begin{array}{l} \frac{1}{24(1-\mu^{2})\sin^{2}\varphi }[(1-\mu )Eh^{3}\cos^{3}\varphi \left(2\frac{\partial^{2}w}{\partial \theta^{2}}+(\cos^{2}\varphi -2)\sin\varphi \frac{\partial v}{\partial \theta }\right)\\ -2\sin\varphi \cos\varphi \frac{\partial^{3}w}{\partial \theta^{2}\partial \varphi }+\cos\varphi \sin^{2}\varphi \frac{\partial^{2}v}{\partial \theta \partial \varphi }+\sin\varphi \cos\varphi \frac{\partial^{2}u}{\partial \theta^{2}}]-\frac{\cos\varphi EhT_{1}{\left(\varphi ,\theta \right)}^{4}}{12-12\mu T_{1}{\left(\varphi ,\theta \right)}^{2}}\\ +\cos\varphi EhT_{1}{\left(\varphi ,\theta \right)}^{4}/[12-12\mu (\cos^{5}\varphi \left(3\frac{\partial w}{\partial \varphi }\sin\varphi -\cos\varphi \frac{\partial^{2}w}{\partial \varphi^{2}}+\cos\varphi \frac{\partial u}{\partial \varphi }-3u\sin\varphi \right)/b^{2})\\ -u\cos^{2}\varphi {\left(\frac{\partial^{2}w}{\partial \theta^{2}}-\sin\varphi \frac{\partial v}{\partial \theta }+\cos^{3}\varphi \sin\varphi \frac{\partial w}{\partial \varphi }-u\cos^{3}\varphi \sin\varphi \right)}^{2}/b^{2}\sin^{2}\varphi ]+\cos^{2}\varphi \sin\varphi \\ =-\frac{\rho hb^{2}\sin\varphi }{\cos^{4}\varphi }(\ddot{u}-\dot{v}\Omega \cos\varphi )\\ \frac{T_{2}(\varphi ,\theta )}{(12-12\mu )b^{2}\sin^{2}\varphi }-\frac{Eh^{3}T_{2}(\varphi ,\theta )}{8(1+\mu )}+\frac{Eh^{3}\cos^{3}\varphi }{24(1+\mu )b^{2}\sin\varphi }[2\frac{\partial^{2}w}{\partial \theta \partial \varphi }-(3-4\cos^{2}\varphi )\frac{\partial v}{\partial \varphi }\sin\varphi \\ -\cos\varphi (3+\sin^{2}\varphi )v+2(\sin^{2}\varphi -\cos\varphi )\frac{\partial^{2}w}{\partial \theta \partial \varphi }-2\cos\varphi \sin\varphi \frac{\partial^{3}w}{\partial \theta \partial \varphi^{2}}\\ -\cos\varphi \sin^{2}\varphi \frac{\partial^{2}v}{\partial \varphi^{2}}-(\sin^{2}\varphi -\cos^{2}\varphi )\frac{\partial u}{\partial \theta }+\cos\varphi \sin\varphi \frac{\partial u}{\partial \theta \partial \varphi }+\cos\varphi T_{2}(\varphi ,\theta )]+T_{3}(\varphi ,\theta )\\ =-\frac{\rho hb^{2}\sin\varphi }{\cos^{4}\varphi }(\ddot{v}+\dot{v}\Omega \cos\varphi +\dot{w}\Omega \sin\varphi )\\ T_{4}(\varphi ,\theta )=-\frac{\rho hb^{2}\sin\varphi }{\cos^{4}\varphi }(\ddot{w}+\dot{v}\Omega \sin\varphi ) \end{array}$$

where *T*_1_ (*φ, θ*), *T*_2_ (*φ, θ*), *T*_3_ (*φ, θ*), *T*_4_ (*φ, θ*) are the complex polynomials about *φ* and *θ*. The motion equation of the radius vector of the point on the resonator in Equation (40) is:

(40) $$\text{R}=\left[U\left(\varphi \right)\cos\left(2\theta \right)\hat{\varphi }+V\left(\varphi \right)\sin\left(2\theta \right)\hat{\theta }+W\left(\varphi \right)\cos\left(2\theta \right)\hat{v}\right]p(t)+\left[U\left(\varphi \right)\sin\left(2\theta \right)\hat{\varphi }-V\left(\varphi \right)\cos\left(2\theta \right)\hat{\theta }+W\left(\varphi \right)\sin\left(2\theta \right)\hat{v}\right]q(t)=\alpha_{1}p(t)+\alpha_{2}q(t)$$

The *N_φ_*, *N_θ_*, *N_v_* are the left form of Equation (39);, the motion equation, *L*, of the bell-shaped resonator circumferential direction is as follows in Equation (41):

(41) $$\text{L}=N_{\varphi }\hat{\varphi }+N_{\theta }\hat{\theta }+N_{v}\hat{v}$$

Substituting the variables into Equation (41), it is derived by the Bubnov-Galerkin method, as follows:

(42) $$\{\begin{array}{c} \int \int_{V}\int \text{L}\alpha_{1}dV=0\\ \int \int_{V}\int \text{L}\alpha_{2}dV=0 \end{array}$$

For the orthogonal curvilinear coordinate system:

(43) $$\{\begin{array}{c} \int_{-\frac{h}{2}}^{\frac{h}{2}}\int_{0}^{2\pi }\int_{\varphi_{b}}^{\varphi_{t}}\left(\text{L}\alpha_{1}\right)R_{\theta }\sin\varphi d\theta R_{\varphi }d\varphi v=0\\ \int_{-\frac{h}{2}}^{\frac{h}{2}}\int_{0}^{2\pi }\int_{\varphi_{b}}^{\varphi_{t}}\left(\text{L}\alpha_{2}\right)R_{\theta }\sin\varphi d\theta R_{\varphi }d\varphi dv=0 \end{array}$$

Simplifying, Equation (43) is as follows in Equation (44):

(44) $$\{\begin{array}{l} \int_{0}^{2\pi }\int_{\varphi_{b}}^{\varphi_{t}}[N_{\varphi }U(\varphi )\cos(2\theta )+N_{\theta }V(\varphi )\sin(2\theta )+N_{v}W(\varphi )\cos(2\theta )]R_{\theta }\sin\varphi R_{\varphi }d\varphi d\theta =0\\ \int_{0}^{2\pi }\int_{\varphi_{b}}^{\varphi_{t}}[N_{\varphi }U(\varphi )\sin(2\theta )-N_{\theta }V(\varphi )\cos(2\theta )+N_{v}W(\varphi )\sin(2\theta )]R_{\theta }\sin\varphi R_{\varphi }d\varphi d\theta =0 \end{array}$$

The dynamic equation of the bell-shaped resonator is derived through the integral operation. However, the equation is too complex to solve. Using the difference method solves this equation, maybe. This work is ongoing.
